# Therapeutics to harness the immune microenvironment in multiple myeloma

**DOI:** 10.20517/cdr.2022.23

**Published:** 2022-06-22

**Authors:** James J. Ignatz-Hoover, James J. Driscoll

**Affiliations:** ^1^University Hospitals, Seidman Cancer Center, Cleveland, OH 44106, USA.; ^2^Department of Biochemistry, Case Western Reserve University, Cleveland, OH 44106, USA.; ^3^Hematopoietic and Immune Cancer Biology Program, Case Comprehensive Cancer Center, Case Western Reserve University, Cleveland, OH 44106, USA.

**Keywords:** Multiple myeloma, drug resistance, proteasome inhibitors, immunomodulators, immunotherapeutics, adaptive resistance, bone marrow microenvironment

## Abstract

Multiple myeloma (MM) remains an incurable, genetically heterogeneous disease characterized by the uncontrolled proliferation of transformed plasma cells nurtured within a permissive bone marrow (BM) microenvironment. Current therapies leverage the unique biology of MM cells and target the immune microenvironment that drives tumor growth and facilitates immune evasion. Proteasome inhibitors and immunomodulatory drugs were initially introduced to complement and have now supplanted cytotoxic chemotherapy as frontline anti-myeloma agents. Recently, monoclonal antibodies, bispecific antibodies, and chimeric antigen receptor T cells were developed to revamp the immune system to overcome immune suppression and improve patient responses. While current MM therapies have markedly extended patient survival, acquired drug resistance inevitably emerges and drives disease progression. The logical progression for the next generation of MM therapies would be to design and validate agents that prevent and/or overcome acquired resistance to immunotherapies. The complex BM microenvironment promotes resistance to both current anti-myeloma agents and emerging immunotherapies. Myeloma cells are intertwined with a complex BM immune microenvironment that contributes to the development of adaptive drug resistance. Here, we describe recently FDA-approved and investigational anti-myeloma agents that directly or indirectly target the BM microenvironment to prevent or overcome drug resistance. Synergistic effects of anti-myeloma agents may foster the development of rationally-designed drug cocktails that prevent BM-mediated resistance to immunotherapies.

## MULTIPLE MYELOMA

Multiple myeloma (MM) is described by clonally expanding plasma cells within the bone marrow (BM), monoclonal proteins detected in blood or urine, and end-organ damage^[1,2]^. Approximately 13% of all hematologic cancers are classified as MM, which is the 2nd most common hematological cancer in high-income and Western countries^[3]^. In the US in 2021, approximately 34,920 new cases of MM (19,320 men, 15,600 women) were reported^[3-6]^.The lifetime risk of an MM diagnosis is 1 in 125 (0.8%). The annual incidence of MM/100,000 persons is 8.2 cases (Caucasian men), 5.0 cases (Caucasian women), 16.5 cases (African-American men), 12.0 cases (African-American women), 8.2 (Hispanic men), 5.7 (Hispanic women) and 5.0 (Asians/Pacific Islander men), and 3.2 (Asians/Pacific Islander women)^[3-6]^. Approximately 12,410 deaths from MM (6,840 men, 5,570 women) were expected in 2021^[4]^. Newly reported cases of MM did not change significantly over the past 10 years, staying in the range of 6.7/100,000 since 2010, while death rates declined slightly, from 3.4/100,000 in 2008 to 3.1/100,000 in 2018^[4]^. Expected 5-year survival has improved to ~56%^[3-6]^. Risk factors include obesity, chronic inflammation, exposure to pesticides, organic solvents, and radiation, and inherited genetic variants^[6-8]^.

MM starts as an asymptomatic precursor condition monoclonal gammopathy of undetermined significance (MGUS) or smoldering multiple myeloma (SMM). Genetic abnormalities, e.g., hyperdiploidy, translocations are already evident in MGUS and SMM^[9-11]^. While these precursors may exhibit a significant burden of clonal plasma cells, they require additional genetic changes to drive end-organ damage and become MM.

Current MM therapy leverages the unique features of plasma cell biology that proliferate within the BM to promote deep clinical remissions with fewer side effects than cytotoxic chemotherapy. The first FDA-approved proteasome inhibitor (PI) bortezomib, immunomodulatory drugs (IMiDs) thalidomide, lenalidomide and pomalidomide (Celgene), and monoclonal antibodies that target CD38 and SLAMF7 have significantly extended patient outcome [Table 1]^[9,12,13]^. These FDA-approved agents are used to treat newly diagnosed patients with related next-generation agents exhibiting activity in the relapsed and/or refractory MM (RRMM) in all stages of treatment^[9,12,13]^. While these agents have markedly improved survival, MM remains incurable, with therapeutic resistance invariably emerging even in patients with an initial favorable response to therapy. Further efforts are needed to define tumor and BM-driven resistance mechanisms to inform next-generation therapies.

**Table 1:** Effects of FDA-approved and investigational agents on the myeloma immune microenvironment

**Therapeutic agent**	**Stage**	**Target**	**Effect on myeloma-immune microenvironment**
**Proteasome inhibitors**			
Bortezomib (Velcade)	FDA-approved	Proteasome 5	Bortezomib inhibits osteoclast differentiation induced by the RANKL, stimulates osteoblast differentiation and inhibits autocrine/paracrine signaling in MSCs and in ECM. PIs also reduce MM adhesion to BMSCs^[15,16,17,24,33-35]^
Carfilzomib (Kyprolis)	FDA-approved	Proteasome 5	
Ixazomib (Ninlaro)	FDA-approved	Proteasome 5	
**IMiDs**			
Thalidomide (Thalomid)	FDA-approved	CRBN	IMiDs promote anti-proliferative, T-cell co-stimulatory, anti-angiogenic and anti-inflammatory effects^[38-44]^
Lenalidomide (Revlimid)	FDA-approved	CRBN	
Pomalidomide (Pomalyst)	FDA-approved	CRBN	
Cel-MODCC-92480	Phase 1/2	CRBN E3 Ub ligase Modulator	Antitumor and immunostimulatory activities
CC-220 (Iberdomide)	Phase 1b/2a	CRBN E3 Ub ligase Modulator (CelMOD)	Antitumor and immunostimulatory activities
C-92480 (Mezigdomide)	Phase 1/2	CRBN E3 Ub ligase Modulator (CelMOD)	Antitumor and immunostimulatory activities
**Monoclonal antibodies**			
Daratumumab (Darzalex)	FDA-approved	CD38	Augments NK-cell cytotoxicity, induces robust increases in helper and cytotoxic T-cell absolute counts. Increases memory T cells while decreasing naïve T cells. Eliminates CD38+ immune suppressor cells, e.g., Tregs, Bregs, and MDSCs^[61-69]^
Elotuzumab (Empliciti)	FDA-approved	SLAMF7	Induces TAM activation and mediates ADCP through an FcγR-dependent manner *in vitro*
Isatuximab (Sarclisa)	FDA-approved	CD38	Eliminates CD38+ immunosuppressive Tregs and alleviates BM-induced immunosuppression
**Nuclear export inhibitors**			
Selinexor (Xpovio)	FDA-approved	Exportin 1, (XPO)	Increased NK cell cytotoxicity in vitro, potentiates ADCC, downregulates pro-survival signals from BM microenvironment, blunts the protective effects from pro-survival signals from TNF, IL-6, IL-4, BAFF BMSCs^[82-87]^
**Bone-modifying agent**			
Denosumab (Prolia)	FDA-approved	RANKL	Potent Inhibitor of osteoclast function^[88-90]^
**Bisphosphonates**			
Zoledronate (Zometa)	FDA-approved	Farnesyl diphosphate	Reduces osteoclast function, inhibits liberation of matrix-synthase (FDPS) bound cytokines, increases IFN-γ production by IL-2-primed NK cells, decrease tumor cell adhesion to bone, and activates T cells^[91-94]^
**CAR T cells**			
Idecabtagene vicleucel (ida-cel, Abecma, bb2121)	FDA-approved	TNFRSF17 (BCMA)	T cells are physically recruited and linked to tumor surface Ags to elicit an anti-tumor immune response and overcome BM microenvironment-mediated immunosuppression^[95-101]^
Ciltacabtagene autoleucel (Carvykti, cilta-cel LCAR-B38M, JNJ-4528)	FDA-approved	Two llama-derived Abs that bind human BCMA	Reduce BCMA-cell expression and microenvironment-mediated immunosuppression
**Bispecific T Cell Engagers**			
Blinatumumab (Blincyto)	FDA-Approved (R/R ALL)	CD19-targeting engager (CD19xCD3)	BiTEs bind simultaneously to T cells and tumor Ags, recruits T cells to tumors and tumor T-cell microenvironment, leading to T cell activation, proliferation, and tumor cell death
	Pilot Study (MM) (NCT03173430)		
Talquetamab	Phase I/II (MM) (NCT03399799)	GPRC5D-targeting bi-specific T-cell engager (GPRC5D x CD3)	Actively kills GPRC5D+ MM cell lines and primary MM cells *in vitro*^[110-115,121]^
AMG420 (NCT03836053)	Phase 1b (RRMM)	BCMA-targeting bi-specific T-cell engager (BCMA x CD3)	Short half-life with encouraging activity in RRMM. Three patients dosed with 400 µg/d had MRD-negative CRs, 2 more responders in the dose confirmation cohort, 3 patients at lower doses attained CRs. No major toxicities were observed up to 400 µg/d^[110-115,122,123]^
Teclistamab (JNJ-64007957)	Phase 1 (MM) (NCT03145181)	BCMA-targeting bi-specific T-cell engager (BCMA x CD3)	At the phase 2 dose, showed promising efficacy and durable responses, well tolerated^[110-115,124]^

Effect of FDA-approved and developmental agents on other cell types within the BM microenvironment. Listed are anti-myeloma agents, putative targets and effects within the myeloma microenvironment. FDA: Federal Drug Administration; IMiD: immunomodulatory drug; NF-κB: nuclear factor kappa-B; MSC: mesenchymal stem cell; ECM: extracellular matrix; BMSC: bone marrow stromal cell; CRBN: Cereblon (CUL4-CRBN E3 Ub ligase complex); Ub: ubiquitin; TAM: tumor-associated macrophage; RANKL: receptor activator of NF-κB ligand; RRMM: relapsed and/or refractory multiple myeloma; BiTE: bispecific T cell engager; SLAMF7: signaling lymphocytic activation molecule F7; TNF: tumor necrosis factor; IFN-γ: interferon-gamma; BAFF: B-cell activating factor; MDSCs: myeloid-derived suppressor cell; ADCP: antibody-dependent cell cytotoxicity; ALL: acute lymphoblastic leukemia; Ags: antigens; BCMA: B-cell maturation antigen; GPRC5D: G protein-coupled receptor, class C group 5 member D.

## MM EFFECTS ON BONE MARROW

Evasion and suppression of antitumor immunity is an essential step in myelomagenesis. MM cells replicate and proliferate nearly exclusively within the BM niche, highlighting the role of the microenvironment in supporting cancer growth^[14-16]^. The BM microenvironment is highly vascularized and consists of a cellular compartment divided into hematopoietic cells, e.g., hematopoietic stem cells (HSC), T and B lymphocytes, myeloid and natural killer (NK) cells, and osteoclasts. The non-hematopoietic cell types include bone marrow stromal cells (BMSCs), osteoblasts, endothelial cells (EC), and fibroblasts^[13-16]^. The non-cellular compartment consists of an extracellular matrix (ECM), oxygen concentration, and a soluble cocktail milieu of growth factors, cytokines and chemokines. Plasma cell clones traffic in and out of the BM to foster metastatic progression while other cell types re-circulate into and out of the BM to promote cytokine-driven myeloma growth^[14-16]^. The tumor-immune microenvironment supports the acquisition of resistance to cytotoxic chemotherapy, biologic agents and immunotherapies leading to immune escape^[17-20]^. Recently it was shown using a pumpless culture platform that adhesion of patient-derived MM cells (PMMCs) to osteoblasts and osteoblast long-term viability were critical factors for ex vivo survival of PMMCs^[21]^. Osteoblasts can also subvert the anti-myeloma effect of NK cells. Since NK cells (and genetically-engineered chimeric antigen receptor-modified NKs) have clinical potential, a better understanding of the osteoblast role as immune regulators in BM is essential^[22]^. Similarly, osteoclasts regulate antigen-dependent T cell activation and responses. Like macrophages, monocytes, and dendritic cells (DCs), osteoclasts display phenotypic and functional plasticity that is dependent on their origin and environment^[23]^.

The highly organized BM integrity is disrupted by the invasion of MM cells. A liquid milieu of cytokines, chemokines, growth factors, and inflammatory mediators mixed with matrix remodeling enzymes enables the communication between tumor, immune and microenvironment cells. Circulating tumor cells, exosomes, cell-free DNA, and apoptotic bodies negotiate the transfer of genetic information from myeloma cells to other tumor and non-tumor cell types^[24-29]^. Exosomes are small, secreted vesicles that confer the bidirectional transfer of proteins, lipids, and nucleic acids between BM and tumor cells. Exosomes can support myelomagenesis by promoting angiogenesis, osteolytic lesions, and drug resistance^[17-21]^. The content of exosomes from MM patients differs from that of healthy donors and could potentially serve as biomarkers and targets.

MM cells are decorated with adhesion molecules that localize myeloma cells to the ECM^[30]^. Collagen I, collagen III, and elastin recently were shown to block the cytotoxic effect of NK cells and promote their production of chemokines and cytokines^[31]^. NK cell cytotoxicity against major histocompatibility complex (MHC)-I-deficient melanoma was markedly increased by blocking tumor collagen deposition. MHC-I down-regulation occurred in solid cancers, which could be directly targeted by circulating cytotoxic NK cells. Prior studies have demonstrated that BMSCs produce paracrine factors and cytokines that drive cell-cell engagement and induce the generation of osteolytic lesions^[32-38]^. Physical interaction between MM and BMSCs, as well as transforming growth factor (TGF) and interleukin (IL)-6 enhance the formation of lytic bone lesions^[32,33]^. Cell-cell interactions and cell adhesion also enhance drug resistance in MM cells^[33-36]^. Unlike healthy mesenchymal stem cells (MSCs), myeloma MSCs enhance tumor survival by producing elevated levels of IL-1β and tumor necrosis factor-alpha (TNF-α)^[33-39]^.

## PROTEASOME INHIBITORS

Proteasome inhibitors (PIs) are the backbone components of current anti-myeloma regimens^[40]^. Bortezomib (Millennium-Takeda) demonstrates clinical efficacy and safety for newly diagnosed and RRMM disease. However, the emergence of chemoresistance and the development of adverse effects, especially peripheral neuropathy, can limit clinical utility. Second-generation PIs carfilzomib (Onyx/Amgen) and ixazomib (Millennium-Takeda) are approved for RRMM and may overcome resistance with better tolerability. Bortezomib has received regulatory approval for intravenous and subcutaneous administration, while ixazomib is the only orally bioavailable PI.

**Figure 1:**
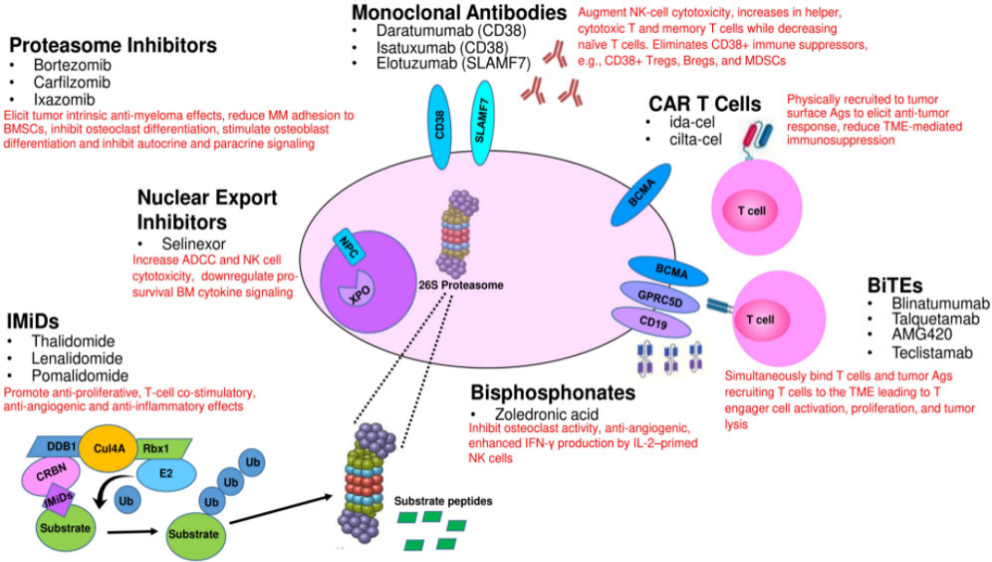
Direct and indirect effects of FDA-approved and developmental agents on MM cells and their interaction with other cell types within the tumor microenvironment. CAR: Chimeric antigen receptor.

PIs also target components of the BM immune microenvironment [Figure 1]^[40,41]^. Kim *et al*. reported that bortezomib impaired BMSCs proliferation *in vitro*^[41]^. PIs downregulate autocrine and paracrine signaling signaled by ECM and MSCs, which impairs myeloma cell growth and survival^[14,42]^. In addition, PIs suppress interleukin-6 (IL-6), IGF-1, and TNF-α production to decrease CXCL12 production by BMSCs^[43,44]^. BM angiogenesis also plays an important role in myelomagenesis and suppresses angiogenesis by decreasing VEGF secretion. Roccaro *et al*. studied MM patient-derived endothelial cells to determine the effects of bortezomib on the angiogenic phenotype^[43]^. At clinically achievable doses, bortezomib inhibited the proliferation of MM patient endothelial cells as well as human umbilical vein endothelial cells in a dose- and time-dependent manner. The binding of MM.1S cells to patient-derived endothelial cells augmented the proliferation of myeloma cells, which was abolished by bortezomib. Bortezomib blocked vascular endothelial growth factor (VEGF) and IL-6 secretion by endothelial cells from myeloma patients and reduced VEGF, IL-6, insulin-like growth factor-I, Angiopoietin (Ang1/Ang2) transcription. Taken together, the results illustrate that bortezomib elicits anti-angiogenic effects in BM.

## IMMUNOMODULATORY DRUGS

Thalidomide, lenalidomide, and pomalidomide (Celgene/Bristol-Myers Squibb) are immunomodulatory drugs (IMiDs) that have contributed to the improvement in MM patient survival^[44]^. Lenalidomide is employed to treat transplant-eligible and ineligible (NDMM) as maintenance post-transplant and for RRMM. IMiDs are thalidomide analogs, which exhibit pleiotropic anti-myeloma activities such as anti-proliferation, anti-angiogenesis, anti-inflammatory, immunomodulatory and cytotoxic effects^[44-47]^. IMiDs also impact the BM microenvironment to lower IL-6 concentrations. Following the introduction of alkylating agents for MM, thalidomide was the next agent to change disease course through VEGF suppression, immunomodulatory and anti-inflammatory effects^[44-46]^. IMiDs increase T cell and NK cell activity, downregulate cytokines, inhibit bone resorption, and decrease cell adhesion molecules (CAM) to disrupt MM-BMSC interactions and IL-6 production^[45-47]^.

Communication between myeloma cells and other cellular components of the tumor microenvironment, e.g., osteoclasts, osteoblasts, and BMSCs, is bidirectional and highly complex. Lenalidomide downregulates hyperactive osteoclasts and reduces the secretion of osteoclastogenic MIP-1a, B-cell activating factor (BAFF), a proliferation-inducing ligand (APRIL), and receptor activator of nuclear factor kappa-B (NF-κB) ligand (RANK-L)^[48,49]^. Lenalidomide has also been shown to more significantly decrease TNF-α, IL-1β, IL-6, and interleukin-12 (IL-12) levels and increases interleukin-2 (IL-2) and IFN-γ production compared to thalidomide^[50]^. LeBlanc *et al*. found that IMiDs co-stimulated T cells through the B7-CD28 pathway^[51]^. IMiDs prolonged T cell priming and boosted the uptake of tumor antigens by DCs to improve the efficacy of antigen presentation^[52,53]^. IMiDs also enhance NK and NK T cell activities^[54]^ and inhibit T regulatory cells (Tregs) proliferation and activity^[55]^. IMiDs decrease IL-2, IFN-γ, and SOCS1 expression in CD4+ T, CD8+ T, NK+ T, and NK cells from peripheral blood (PB) and B^[56]^.

Programmed death (PD)-1 and PD-ligand-1 (PD-L1) interactions attenuate the production of cytotoxic T lymphocytes (CTLs) that recognize tumor cells. PD-L1 expression on plasma cells from MM patients is markedly upregulated compared to those from MGUS patients and healthy volunteers^[57]^. IMiDs downregulate PD-1 levels on T and NK cells and PD-L1 on myeloma cells to promote antibody-dependent cellular cytotoxicity (ADCC). Bortezomib and lenalidomide do not have the flexibility to subdue myeloid-derived suppressor cell (MDSC) activity, whereas CD38-targeting agents do have this capacity^[58,59]^. Co-stimulatory effects of IMiDs on T and NK cells have been proposed to enhance anti-MM immunity but are yet to be demonstrated *in vivo*.

## MONOCLONAL ANTIBODIES

The introduction of PIs and IMiDs represented an initial paradigm shift in MM treatment strategy. Subsequently, in 2015 two monoclonal antibodies were FDA-approved for RRMM treatment and represented a second shift in the treatment approach towards immunotherapies. Daratumumab (Janssen Oncology) is a humanized monoclonal IgG-κ antibody that binds to the transmembrane glycoprotein CD38 (cyclic ADP ribose hydrolase)^[60]^. CD38 is expressed on immune cells, overexpressed on myeloma cells, and contributes to cell adhesion and ecto-enzymatic activities. Daratumumab binds CD38, causing cells to undergo ADCC, complement-dependent cytotoxicity (CDC), and antibody-dependent cellular phagocytosis (ADCP). Combination regimens incorporating daratumumab have demonstrated promising results in the relapsed refractory setting and are increasingly used upfront and in transplant-eligible patients^[61-64]^. Phase III trials showed promising results when daratumumab was combined with lenalidomide and dexamethasone, with bortezomib and dexamethasone, and, in quadruplet therapy with bortezomib, dexamethasone, and lenalidomide^[61-64]^.

Daratumumab also targets CD38+ immune, non-myeloma cell populations. PB and BM were obtained and analyzed before and during therapy and at relapse from RRMM patients enrolled in two daratumumab monotherapy studies^[65]^. CD38-expressing regulatory B cells (Bregs) and MDSCS were evaluated to determine the effect of daratumumab on immunosuppressive activity. A unique subpopulation of CD38+ Tregs was found to be more immunosuppressive than CD38- Tregs *in vitro* and was reduced in daratumumab-treated patients. Likewise, daratumumab treatment generated significant elevations in helper and cytotoxic T-cell absolute counts. In PB and BM, daratumumab induced significant increases in CD8+:CD4+ and CD8+:Treg ratios and increased memory T cells while decreasing naïve T cells. The majority of patients demonstrated broad T-cell changes, although patients with a partial response or better showed greater maximum effector and helper T-cell increases. Greater increases in T-cell clonality, measured by T-cell receptor (TCR) sequencing, positively correlated with increased CD8+ PB T-cell counts. Depletion of CD38+ immunosuppressive cells, which is related to a rise in T-helper cells, cytotoxic T cells, T-cell functional response, and TCR clonality, represents an additional mechanism of action for daratumumab and deserves further exploration. The anti-myeloma benefit of daratumumab can be potentiated when combined with bortezomib which leads to increased expression of CD38 target on MM cells. However, daratumumab may also internalize CD38 in MM cells to inhibit adhesion to BMSCs and overcome CAM drug resistance (CAM-DR)^[66]^.

The second humanized monoclonal FDA-approved for MM is elotuzumab (Bristol-Myers Squibb), which binds the signaling lymphocytic activation molecule family 7 (SLAMF7, CD319, cell-surface glycoprotein CD2 subset 1/CS1), on the MM cell surface^[67]^. SLAMF7 is also modestly expressed on NK cells and certain T cells^[68,69]^. In combination with lenalidomide and dexamethasone, elotuzumab enhances progression-free survival (PFS) in RRMM.

Awwad *et al*. demonstrated that SLAMF7 was expressed at high levels on CD8+CD28-CD57+ Tregs from MM patients^[70]^. SLAMF7 levels were also linked with the expression of T cell exhaustion transcription factor signatures and cell surface markers. Elotuzumab specifically depleted SLAMF7+CD8+ T cells *in vitro* and *in vivo* through macrophage-dependent ADCP. SLAMF7 may serve as an indicator to identify CD8+ Tregs and anti-SLAMF7 antibodies that enhance anti-myeloma responses.

Isatuximab (Sanofi/Genzyme) targets a specific epitope on the transmembrane glycoprotein CD38, different from that targeted by daratumumab, and inhibits CD38 hydrolase activity^[68-75]^. CD38 regulates migration and receptor-mediated adhesion by binding to CD31 or hyaluronic acid. Isatuximab induces myeloma death through fragment crystallizable (Fc)-dependent mechanisms, e.g., ADCC, ADCP, and CDC, and direct Fc-independent mechanisms^[72]^. Isatuximab downregulates constitutive and inducible Tregs resulting in enhancing the anti-myeloma response of other immune cell types^[73-75]^.

Isatuximab was evaluated as monotherapy and demonstrated promising results in a phase I study of 35 RRMM patients as well as a subsequent phase II study alone and in combination with dexamethasone in heavily-pretreated patients^[76,77]^. Isatuximab added to a pomalidomide-dexamethasone regimen improved PFS, which represents another option to treat lenalidomide- and PI-refractory disease^[78]^. When combined with pomalidomide and low-dose dexamethasone, isatuximab led to improved PFS and a 40% reduction in the risk of disease progression or death. Patients had an overall response rate (ORR) of 60.4%, compared to 35.3% for patients who only received pomalidomide and dexamethasone. Isatuximab demonstrated a statistically significant increase in TCR clonality after treatment compared to that at treatment initiation, suggesting that isatuximab increases host antitumor immunity.

A recent study assessed isatuximab in heavily-pretreated RRMM patients despite receiving prior anti-CD38 therapy, most patients having been recently exposed to daratumumab combination therapy^[79]^. Most patients (77%) experienced a response of MR or better with isatuximab. While objective responses were not observed, one patient achieved MR and 17 patients had stable disease as the best overall response^[80]^. A prospective, randomized, open-label, phase 3 trial compared isatuximab combined with carfilzomib-dexamethasone to carfilzomib-dexamethasone in relapsed MM patients^[81]^. Isatuximab addition significantly improved PFS and depth of response, representing a new standard of care for this group.

Daratumumab, elotuzumab, and isatuximab act by recruiting immune effectors to enhance cellular cytotoxicity directed against myeloma cells. The anti-myeloma activity of daratumumab and elotuzumab appears independent of the disease stage. These agents may adversely generate allergic-type infusion reactions. Potential complications in serum protein electrophoresis testing and daratumumab cross-reactivity with CD38 present on erythrocytes should be considered. The success of daratumumab and elotuzumab in RRMM has ignited enthusiasm for the development of additional CD38-targeting agents. To note, hypoxia within the BM microenvironment suppresses the maturation of MM cells as well as the expression of CD38 and SLAMF7. While antibody therapy was initially approved for RRMM, there is interest in incorporating monoclonal antibodies into conditioning regimens for NDMM as well.

## NUCLEAR EXPORT INHIBITORS

Selinexor (Karyopharm Therapeutics) is a first-in-class orally bioavailable drug that inhibits the nuclear export protein exportin1 (XPO1)^[82]^. Selinexor has been FDA-approved for use combined with dexamethasone and bortezomib in MM patients previously treated with four prior therapies, including at least two PIs and at least two IMiDs^[82,83]^. XPO1 overexpression is linked with a worse prognosis in solid tumors and blood cancers^[84]^. Selinexor also demonstrates an ability to modulate tumor immunology and the surrounding tumor microenvironment. Treatment of B cell lymphomas with selinexor led to increased NK cell-mediated cytotoxicity *in vitro *and selinexor potentiated ADCC-mediated by rituximab and obinutuzumab^[85]^. NK cells exhibited greater IFN-γ and CD107a expression, both activities associated with NK activation, and lymphoma cells downregulated HLA-E, which binds the inhibitory NKG2A receptor. Zhong *et al*. demonstrated that selinexor may also downregulate pro-survival signals that originate from the BM microenvironment^[86]^. Treatment of CLL cells with selinexor blunted protective effects from anti-apoptotic, pro-survival signals from TNF, IL-6, interleukin-4 (IL-4), BAFF, and CD40L *in vitro *and also blunted anti-apoptotic effects of marrow-derived fibroblast co-culture model. Selinexor may help overcome hypoxia-mediated PI resistance *in vitro *as well and restore PI sensitivity *in vivo*^[87]^.

## DENOSUMAB

The receptor activator of nuclear factor-κB ligand (RANKL)/RANK signaling system modulates osteoclastogenesis leading to bone resorption^[88]^. Denosumab (Amgen) is a humanized monoclonal antibody that neutralizes RANKL, inhibits osteoclasts, and decreases the rate of skeletal-related events not only in MM but also in solid tumors^[89,90]^. Denosumab treatment can inhibit the RANKL/RANL receptor interaction and suppress osteoclastic bone resorption.

## BISPHOSPHONATES

Zoledronate (Novartis) and pamidronate (Novartis) are pyrophosphate analogs (bisphosphonates), which demonstrate a high affinity for bone and the capacity to impair osteoclast function as well as anti-angiogenic activities^[91-93]^. Bisphosphonates inhibit farnesyl pyrophosphate synthase and reduce isoprenylation of Rab, Rac, and Rho. The bisphosphonates are provided as supportive therapy in MM since they are associated with lower rates of vertebral fractures, reduced skeletal-related events, and decreased pain but are associated with an increased risk of jaw osteonecrosis. Nussbaumer *et al*. showed that zoledronic acid enhanced IFN-γ production by IL-2-primed NK cells in CD14+CD56+ DC-like cell-dependent process that may also require γδ T cells^[94]^.

## CAR T CELLS AND BISPECIFIC T CELL ENGAGERS

The adoptive transfer of chimeric antigen receptor (CAR)-expressing T cells is a transformative approach to improve cancer treatment. B-cell maturation antigen (BCMA) displays restricted RNA expression and is selectively expressed by B-lineage cells. BCMA is not detected in healthy tissues and was not detected on human CD34+ HSCs^[95]^. T cells expressing a CAR-targeting BCMA had substantial activity against heavily-treated RRMM patients^[96]^. BCMA-targeted CAR T-cell therapies that differ in the costimulatory domain demonstrate efficacy in early-phase trials^[97,98]^. In 2021, the FDA approved idecabtagene vicleucel (ide-cel, Bristol-Myer Squibb), a BCMA-directed genetically modified autologous T cell therapy, for MM patients that had not responded to > 4 different prior treatments^[99]^. In 2022, ciltacabtagene autoleucel (cilta-cel), a CAR T-cells with 2 BCMA-targeting single-domain antibodies, was evaluated in RRMM patients with poor prognosis^[100]^. A single infusion of cilta-cel yielded early, deep, and sustained responses in heavily pretreated patients leading to regulatory approval. CAR T cell therapies have limitations that include life-threatening toxicities, modest antitumor activity, antigen escape, restricted trafficking, and limited tumor infiltration^[101-103]^. The ECM is composed of fibrous glycosaminoglycans and proteoglycans that act as a physical barrier to CAR T cells and prevent their penetration and infiltration of tumors. Matrix-degrading agents that improve immune cell infiltration may enhance the efficacy of CAR-T cells^[104-108]^.

Bispecific T cell engagers (BiTEs) are novel antibody constructs targeting T cells to a tumor antigen. The prototypical BiTE- blinatumumab (Glaxo-Smith Kline) targets CD3 and CD19 to facilitate T cell-mediated killing of relapsed acute lymphocytic leukemia (ALL) cells [Table 1]^[109,110]^. BiTEs may promote downregulation of their target antigen as a mechanism of immune escape, as evidenced by a metanalysis of ALL patients initially treated with blinatumomab exhibiting increased relapse after CAR T therapy and decreased event-free survival with a trend towards exhibiting more CD19 dim disease. Thus, the development of BiTEs and CAR-T cells with differing target ligands is of clinical interest. Multiple BiTEs have shown promising results in MM including several anti-BCMA/CD3 conjugates as well as talquetamab (Janssen Pharmaceutical Companies of Johnson & Johnson), an anti-GPRC5DxCD3 conjugate that targets endogenous T cells to MM cells with a less severe side effect profile than CAR-T cells with step-up outpatient dosing that can be given to the transplant-ineligible patients^[111-113]^. The immunosuppressive nature of BMSCs poses a significant hurdle to anti-myeloma immunotherapies. Recently, it was shown that MM or AML cell co-culture with the stromal cell lines HS-5 and HS-27a protected the tumor cells from bispecific antibodies that target CD123 and BCMA^[114]^. The reduction in T cell effector responses was correlated with impaired CD3 redirection cytotoxicity. Cell-cell contact of tumor cells with stromal cells was thought to decrease T cell activation. Agents that inhibit the very late antigen 4 (VLA4) adhesion pathway may be combined with CD3 redirection to reduce stroma-mediated inhibition of T cell activation. The results lend support to inhibiting VLA4 functional activity as well as administering CD3 redirection therapeutics as a combinatorial regimen that enhances antitumor immunity.

## CONCLUSIONS AND FUTURE PERSPECTIVES

The introduction of IMiDs demonstrated the clinical value of immunotherapeutic approaches for the treatment of MM. However, BM-mediated therapeutic resistance promotes tumor escape and immune evasion that represent obstacles to extending patient outcomes. Recently developed myeloma-directed immunotherapies, e.g., monoclonal antibodies, CAR-T cells, antibody-drug conjugates (ADCs), and BiTEs represent the emerging phase of myeloma care^[115-118]^. Similar to the mechanisms of resistance observed following the administration of cytotoxic chemotherapy, PIs and IMiDs, novel strategies are needed to prevent or overcome resistance to immunotherapies. Numerous immune cell types, e.g., Tregs, Bregs, MDSCs, macrophages, dysfunctional DCs, MSCs, osteoclasts, as well as the ECM, modulate and suppress T- and NK cell activity. BM-mediated immune exhaustion as well as immune checkpoint proteins on T- and NK cells and their corresponding ligands on MM cells, e.g., PD1/PDL-1, or T cell immunoglobulin and tyrosine-based inhibitory motif (TIGIT) domains, represent additional obstacles^[119-121]^. Innovative platforms will provide the foundation for the next paradigm shift in myeloma to overcome current limitations and improve high-risk and newly diagnosed patient survival^[115-117]^.

## DECLARATIONS

### Copyright

© The Author(s) 2022.
